# Monitoring the molecular composition of live cells exposed to electric pulses via label-free optical methods

**DOI:** 10.1038/s41598-020-67402-x

**Published:** 2020-06-26

**Authors:** Antoine Azan, Marianne Grognot, Tomás García-Sánchez, Lucie Descamps, Valérie Untereiner, Olivier Piot, Guilhem Gallot, Lluis M. Mir

**Affiliations:** 1 Vectorology and Anticancer Therapies, UMR 8203, CNRS, Gustave Roussy, Univ. Paris-Sud, Université Paris-Saclay, 114 rue Edouard Vaillant, 94805 Villejuif, France; 2 Laboratoire D’Optique Et Biosciences, Ecole Polytechnique, CNRS, INSERM, IP Paris, 91128 Palaiseau Cedex, France; 3 MeDIAN, Biophotonics and Technologies for Health, MEDyC, UMR 7369, CNRS, University of Reims Champagne-Ardenne, 51 rue Cognacq-Jay, 51096 Reims, France; 4 Cellular and Tissular Imaging Platform PICT, Faculty of Pharmacy, University of Reims Champagne-Ardenne, 51 rue Cognacq-Jay, 51096 Reims, France; 5 Metabolic and Systemic Aspects of Oncogenesis (METSY), CNRS, Institut Gustave Roussy, Université Paris-Saclay, 94805 Villejuif, France

**Keywords:** Membrane biophysics, Membrane biophysics, Raman spectroscopy, Raman spectroscopy

## Abstract

The permeabilization of the live cells membrane by the delivery of electric pulses has fundamental interest in medicine, in particular in tumors treatment by electrochemotherapy. Since underlying mechanisms are still not fully understood, we studied the impact of electric pulses on the biochemical composition of live cells thanks to label-free optical methods: confocal Raman microspectroscopy and terahertz microscopy. A dose effect was observed after cells exposure to different field intensities and a major impact on cell peptide/protein content was found. Raman measurements reveal that protein structure and/or environment are modified by the electric pulses while terahertz measurements suggest a leakage of proteins and other intracellular compounds. We show that Raman and terahertz modalities are a particularly attractive complement to fluorescence microscopy which is the reference optical technique in the case of electropermeabilization. Finally, we propose an analytical model for the influx and efflux of non-permeant molecules through transiently (electro)permeabilized cell membranes.

## Introduction

The main consequence of the delivery of high intensity pulsed electric fields (PEF) of very short duration on biological samples is the permeabilization of the plasma membrane^1^. This interaction between PEF and biological samples, termed electropermeabilization or electroporation, has led to many applications in industry or medicine, in particular using 100 microsecond pulses (µsPEF). For instance, electrochemotherapy consists in the combination of tumor cells electropermeabilization and a chemotherapeutic agent such as bleomycin or cisplatin drugs^2,3^. Electrochemotherapy is nowadays commonly used for the treatment of many cancer types: skin cancer^4^, breast cancer^5^, head and neck cancer^6^, pancreatic cancer^7^, etc. Although electropermeabilization has been studied for decades, the underlying mechanisms are still not fully understood. It is well established that the electric field induces an additive transmembrane potential to the resting transmembrane potential of cells^8^. Molecular dynamics simulations^9,10^ have demonstrated that the creation of pores into the membrane is a stochastic phenomenon: the increase of the external field amplitude increases the probability of formation of the pores. Therefore, to ensure that pores occur within the duration of the applied pulses, appropriated field amplitudes must be used, a situation that led to the definition of functional threshold amplitudes by the experimentalists. The destabilization of the cell membrane is also associated with long-term effects on the membrane, such as membrane disorder and a decrease of membrane elasticity,^11,12^ that occur minutes after the PEF delivery. Recently, we considered biochemical modifications of the membrane as the major contributor to the electropermeabilization process. Mass spectrometry analysis of the chemical composition of a simple membrane model, Giant Unilamellar Vesicles (GUV), exposed to PEF showed the peroxidation of phospholipids induced by the delivery of electric pulses^13^. This chemical damage hypothesis is supported by numerical models^14,15^ and experiments^16,17^. Probing the molecular composition of live cells seems to be a critical point to better understand the interaction between PEF and biological cells. In this study, we used confocal Raman microspectroscopy (CRMS) and terahertz attenuated total reflection (THz-ATR) to monitor biochemical consequences of the interaction between PEF and live cells. These two label-free and non-invasive optical techniques have the major advantage to provide detailed information about the intrinsic molecular composition of the sample.

Combining Raman spectroscopy and confocal microscopy, CRMS gives access to the vibrational footprint of the sample which is related to its intrinsic biochemical composition^18^. CRMS allows to extract information about lipids, proteins and DNA of biological samples. CRMS is now commonly used to characterize cells^19^ and tissues^20^. In recently published studies, our group has demonstrated the interest to use CRMS to investigate the electropermeabilization process^21,22^. The terahertz domain (1 THz = 10^12^ Hz) lies between the infrared and microwaves electromagnetic domains. Thanks to the low associated photon energy (meV), terahertz sensing is a non-invasive, non-disruptive technique. Despite technology restrictions that, for a long time, have limited the use of terahertz to the study of either single purified molecules or simplified and/or pretreated biological structures^23,24^, the terahertz domain has shown to have potential for biomedical applications, despite strong absorption by water^25^. In this field, the most important feature is the direct sensitivity of terahertz radiation to the amount of water in the tissues, but also to the nature and amount of the dissolved solutes^26^. Recent studies demonstrated the possibility to detect and spectroscopically investigate complex systems as living cells^27,28^ and even in vivo accessible tissues^29^. The wavelength of the terahertz waves (1 THz corresponds to a wavelength of 300 µm) also allows, in attenuated total reflection setups, the production of evanescent waves with characteristic length matching the length of the cells^30^. Recently, we demonstrated the ability to monitor the chemical permeabilization of live cells via terahertz microscopy^31^. Finally, a recent study is the only paper to our knowledge dealing with the feasibility to use transmission terahertz spectroscopy to show that changes are produced on cells exposed to electroporation pulses^32^, but without real time monitoring of these changes.

Based on two previous publications of our groups that validated the use of CRMS and THz-ATR techniques to monitor permeabilization^21,31^, we investigate in the present study the effects of different electric field magnitudes on the biochemical composition of live cells exposed to µsPEF. Furthermore, for the first time, THz-ATR measurements allowed real time observation of the cytosol concentration dynamics after µsPEF delivery. The study was conducted on human adipose-derived Mesenchymal Stem Cells (haMSC) and Madin–Darby Canine Kidney (MDCK) cells for the Raman and the terahertz experiments respectively. haMSC are very large cells (~ 70 μm diameter for attached cells) thus enabling an easy access to acquire the Raman signature of the cytoplasm-only area. MDCK cells have the major advantage to easily grow on the specific THz-ATR substrate, which is necessary to have high cell density for the THz-ATR experiments. CRMS and THz-ATR data were compared to fluorescence measurements which are considered as the reference method to characterize cell electropermeabilization through the quantification of the internalization of non-permeant dyes such as YO-PRO-1 or Propidium Iodide (PI)^33^. We employed 100 µs duration pulses since it is a widely used electroporation condition in human and veterinary oncology, as well as in cardiac treatments. Based on this comparative approach, we report here that Raman and terahertz techniques exhibit unique features compared to fluorescence microscopy, bringing complementary information on the electropermeabilization process.

## Materials and methods

### Cell culture

For this study, two different cell types were used. MDCK cells were used in the case of THz-ATR whereas haMSC cells were used in the case of Raman. The permeabilization of both cell lines was also studied by fluorescence microscopy. The selection of the cell lines was based on our previous published work with MDCK cells for the terahertz experiments^31^ and haMSC for the Raman experiments^21^. The choice of these cells was dictated to make easier any comparison with our previous studies. In no case, the choice of the cells was dictated because of a specific sensitivity of the cells to one or the other of the technologies investigated during the work here reported. Both cell types were grown in a humidified atmosphere at 37 °C and 5% CO_2_ in Dulbecco’s Modified Eagle Medium (DMEM) supplemented with 10% serum (fetal calf serum for MDCK and fetal bovine serum for haMSC) and 1% penicillin–streptomycin (Life Technologies).

For the terahertz experiments, the MDCKs were plated at a density of 30,000 cells/cm^2^ on a 3 mm thick 37 mm diameter high-resistivity silicon (HR-Si) window, which is the optimal substrate for terahertz experiments in attenuated total internal reflection geometry^30,31^, and placed in a 60 mm Petri dish. Cells were grown for 3 to 5 days until full confluence. Then, part of the cells was removed by scratching them from one half of the HR-Si window that was used as the reference. Before the experiments, the culture medium was replaced by 2 mL of a buffered minimal medium (HBSS with 10 mM HEPES buffer, without Ca^2+^). Terahertz experiments were performed at room temperature (21 °C), where the terahertz signal was verified to be stable for more than 4 h in control experiments without µsPEF.

Due to the opacity to visible light of the HR-Si supports, which makes them not compatible with standard microscopy, for the fluorescence experiments used for comparing to the terahertz experiments, the cells were prepared exactly under the same conditions but were grown in a standard 35 mm Petri dishes. No cell morphology or proliferation difference was noticed between the culture on the HR-Si windows and the Petri dishes. These fluorescence microscopy experiments were also performed at room temperature (21 °C).

For the Raman experiments, the haMSCs cells were plated at a density of 5000 cells/cm^2^ on a CaF_2_ support (Crystran, Poole) placed at the bottom of a 35 mm Petri dish. Cells were grown overnight. Before the Raman experiments, the medium was replaced by 2 mL of a saline solution, NaCl 154 mM (B. Braun). This saline solution is commonly used for CRMS acquisitions on living cells^34^. Due to the long duration of Raman acquisitions, they were performed at 4 °C to avoid the interference of the effects of membrane resealing process^22^. For the fluorescence experiments to be compared to the Raman experiments, the cells were grown exactly under the same conditions and the recordings were performed at room temperature (21 °C).

### Pulse generator and pulses conditions

A commercially available electric pulses generator (Cliniporator™, IGEA, Italy) was used to treat the cells with 8 pulses of 100 µs delivered at a repetition frequency of 1 Hz. The magnitude of the electric field was 500, 750, 1000, 1250 or 1500 V/cm depending on the experiment. To deliver the electric pulses on attached cells under the different imaging systems, a homemade system of electrodes was used based on two stainless steel parallel plate electrodes separated by a fixed distance of 4 mm or 8 mm for the Raman and terahertz experiments, respectively. The 8 mm distance between the electrodes was chosen in order to prevent any perturbation with the 2.5 mm diameter illumination spot of the THz-ATR beam. Because the maximum output voltage of the pulse generator was 1000 V, the distance of 8 mm between the plate electrodes restricted the maximum electric field magnitude delivered to 1250 V/cm. Therefore, the electric field magnitude of 1500 V/cm was only used for the Raman experiments. The Cliniporator was connected to the plate electrodes with alligator clips. The delivery of the electric pulses was performed at 4 °C for Raman experiments, and at room temperature (21 °C) for the rest of experiments (fluorescence and terahertz). As mentioned in our previous work, no heating effect, pH change or bubble formation was noticed after the delivery of the µsPEF^21^.

### CRMS and spectra processing

The Raman experiments were performed under the experimental conditions detailed in Azan et al*.*^21^. Briefly, a confocal Raman microspectrometer LabRam ARAMIS (Horiba Jobin Yvon) with a 532 nm continuous-wave laser was used to acquire the Raman spectra of living haMSC cells. The power at the sample was around 20 mW which is known to be non-toxic for the cells^35–37^. Prior to any measurement, the confocal Raman microspectrometer was calibrated with a Silicon sample using the 520 cm^−1^ band and the laser power was checked. The Raman signatures were acquired in the Finger Print region (600–1800 cm^−1^). The acquisition time was fixed to two accumulations of 30 s (60 s in total). The sample was placed on an XY piezoelectric stage to investigate multiple locations. During the Raman measurements, cells were maintained at 4 °C by the T95 temperature controller (Linkam Scientific Instrument Ltd). The Raman signature of the saline solution was acquired in order to be able to remove this background signal from the measured spectra. In total, 264 Raman spectra were collected.

The measured Raman spectra were pre-processed as detailed in Azan et al*.*^21^. Briefly, a quality check was performed on each individual Raman spectrum collected, meaning that the measured spectra with a signal to noise ratio (SNR) lower that 10 were discarded from the data set. Then, the spectra were smoothed using the Savitzky-Golay filter (12 points, 2nd order polynomial) and the baseline and the saline solution background signals were removed. Finally, the spectra were normalized using the standard normal variance (SNV) method. The mean normalized spectra for each electric pulse condition were calculated. The difference between the mean normalized spectra at a specific electric pulses condition and the mean normalized spectra of control (sham exposure, 0 V/cm) was also calculated. Multivariate analysis, based on Partial Least Squares (PLS) was performed on the mean-centered combined data set. The magnitude of the electric field was used as the observable variable for PLS analysis. Latent Variable 1 (LV1) scores obtained from the analysis were used in subsequent data processing.

### Terahertz-attenuated total reflection and specific data processing

The terahertz signal was generated and detected by a classical Terahertz time-domain spectroscopy (THz-TDS) setup^30^. This setup generates an almost linearly polarized sub-single cycle terahertz pulse, centered around 0.5 THz and extending up to 2 THz. As a signal, the maximum amplitude of the terahertz pulse is chosen as it demonstrates the strongest modification between cells and free patch surface. In addition, the terahertz attenuated total reflection (THz-ATR) device is a completely terahertz-transparent HR-Si isosceles prism (R > 10 kΩ cm, n = 3.42) with a base angle of 42°. This incident angle provides total internal reflection conditions. For imaging the cells, the silicon prism was topped with the 3 mm thick HR-Si window on which cells had been previously grown. An additional ring cover enabled to maintain cells in their buffered medium and to move very precisely the substrate. Under such conditions, the cell layer was probed by an evanescent wave of a longitudinal extension of about 20 μm generated at the surface of the HR-Si window^30^. Support displacement allowed the acquisition of images, pixel by pixel, with a lateral resolution of 2.5 mm. Acquisitions were made with the silicon patch half covered with a cell layer and half-cell-free as a reference, the whole bathing in cell medium. For the kinetic changes observed after the electric pulses delivery, reported in this article, the signals of three pixels in the cell layer region and two in the reference region were acquired along a line in approximately 15 s to obtain the THz-ATR relative signal value. This signal was normalized by its value before the delivery of electric pulses to obtain the normalized THz-ATR relative signal, Δ_THz_ in percent. Without any perturbation, the THz-ATR signal was stable for hours at room temperature (21 °C). The mentioned relative THz-ATR signal was acquired every 30 s for 40 min after the electric pulses delivery, optimized according to the ATR-THz setup and signal dynamics. The variations in the recorded terahertz signals from cell to reference areas originate from changes of the molecules cytosolic concentrations. More precisely, the THz-ATR relative signal difference between the cells and their outer medium is proportional to the mass concentration of all intracellular molecules, from ions and metabolites to proteins^31,38^ (see also Supplementary Information). This leads to a relative difference of about 8%. We also tested the effect of terahertz radiation on cells viability by putting the cells back in culture after exposure to the terahertz radiation in the absence of µsPEF delivery: the cells grew normally for the next 24 h.

### Fluorescence microscopy (including data pre-processing and processing)

In all the fluorescence experiments, Hoechst 33,342 was used to stain the cell nucleus in order to localize all the cells, permeabilized or not. YO-PRO-1 was used as a classical fluorescence marker of cell electropermeabilization^32^. Prior to the fluorescence experiments, cells were stained in the presence of 370 nM of Hoechst 33,342 for 30 min at 37 °C and 5% CO_2_. After two washes with phosphate-buffered saline (PBS), YO-PRO-1 was added to cells at a final concentration of 1 µM. The same buffers and the rest of specific experimental conditions (substrates, electrodes, buffer volume, etc.) used respectively, for Raman and terahertz experiments were also used in fluorescence experiments and they were performed at room temperature. Fluorescence and bright-field images were acquired with an Observer Z1 inverted microscope (Zeiss). Images were acquired with a fixed exposure time of 300 ms for both the green (YO-PRO-1; λ_ex_ = 475 nm, λ_em_ = 530 nm) and the blue (Hoechst 33,342; λ_ex_ = 365 nm, λ_em_ = 445 nm) channels and 40 ms for the bright-field channel. The microscope was controlled by the Zen Blue 2 Zeiss software.

In the case of the comparison between fluorescence and Raman modalities, images were acquired 10 min after the electric pulses delivery. Subsequently, the YO-PRO-1 fluorescence intensity (I_Fluo_) was automatically extracted from cell nuclear location labeled by the Hoechst 33,342 dye.

In the case of the comparison between fluorescence and THz-ATR methods, the two-channel fluorescence images were acquired every 20 s for at least 20 min, optimized according to the setup and signal dynamics. 4 images were acquired before delivering the electric pulses. The only difference between the THz-ATR experiments and the time-lapse fluorescence experiments was the substrate: the HR-Si used for THz-ATR experiments was replaced by a standard cell-culture Petri dish for the fluorescence experiments. The difference of permittivity of silicon (11.7) and plastic (2.2) has no effect on the electric field distribution generated by the electrodes since the cell medium is conductive. All the other experimental parameters or sample preparation processes remained strictly the same. At the end of the fluorescence experiments, no cell morphology modification was noticed. Additionally, cells grew normally if they were put back in culture, showing absence of phototoxicity. Under these experimental conditions we also checked the absence of YO-PRO-1 and Hoechst 33,342 photobleaching. In each fluorescence image, we selected 5 regions of interest (ROI) including cells and averaged the fluorescence signals over these ROI to obtain the total cell YO-PRO-1 fluorescence signal $F_{cell}$. As well, we selected a background ROI without cell, for the reference YO-PRO-1 signal $F_{ref}$. In order to compensate for the time variations of the lamp used in the fluorescence measurements, we calculated the normalized ratio $S_{fluo} = F_{cell} /F_{ref}$. The two fluorescence signals varying linearly with the intensity of the lamp, $S_{fluo}$ was then independent on the intensity. However, this can possibly lead to a larger unnecessary fluctuation of the ratio when the $F_{ref}$ is very low, compared to a method using the difference $F_{cell} - F_{ref}$. This is fortunately not the case in our measurements, since $F_{ref}$ is far above the detection noise of the camera. The normalization ratio method gave the best result, by strongly limiting the impact of the light power fluctuation in the quantification of the fluorescence intensity, without generating additional fluctuations. Finally, the fluorescence relative signal $I_{Fluo} \left( t \right)$ was calculated as the relative variation between $S_{fluo} \left( t \right)$ after the delivery of the electric pulses and $S_{fluo}$ (t < 0) before the delivery, as

$$ I_{Fluo} \left( t \right) = \frac{{S_{Fluo} \left( t \right) - S_{Fluo} \left( {t < 0} \right)}}{{S_{Fluo} \left( {t < 0} \right)}}. $$

### Data process to compare the different instrumentations

In order to perform a quantitative and qualitative comparison between the different modalities (Raman vs fluorescence and terahertz versus fluorescence), different parameters were defined. The normalized relative Raman signal Δ_Raman_ and the normalized fluorescence signal Δ_Fluo_ were used to compare Raman and fluorescence modalities. Similarly, the time-evolution of the normalized relative THz-ATR signal Δ_THz_(t) and the time-evolution of the normalized relative fluorescence signal Δ_Fluo_(t) were used to compare THz-ATR to fluorescence modality. These parameters were defined by the following equations

$$ \Delta_{Raman } = \frac{{I_{Raman} - { < }I_{Raman\;Control} { > } }}{{{ < }I_{Raman \;Control} { > }}} $$

where I_Raman_ was the LV1 score and <I_Raman Control_> was the mean LV1 score of the control group (0 V/cm),

$$ \Delta_{Fluo } = \frac{{I_{Fluo} - { < }I_{Fluo\;Control} { > } }}{{{ < }I_{Fluo\;Control} { > }}} $$

where I_Fluo_ was the YO-PRO-1 normalized fluorescence intensity per cell and <I_Fluo Control_> was the mean YO-PRO-1 normalized fluorescence intensity of the control group (0 V/cm), and

$$ \Delta_{THz } \left( t \right) = \frac{{S_{THz} \left( t \right) }}{{S_{THz} (t < 0)}} $$

where

$$ S_{THz } \left( t \right) = \frac{{E_{THz} \left( {{\text{cell}}} \right) - E_{THz} \left( {{\text{buffer}}} \right) }}{{E_{THz} \left( {{\text{buffer}}} \right)}} $$

and where $E_{THz}$ is the peak amplitude of the reflected THz-ATR signal from the ATR device.

In the case of THz-ATR experiments, Δ_Fluo_(t) and Δ_THz_(t) were fitted with the following exponential functions ${\Delta }_{Fluo} { }\left( {\text{t}} \right) = C_{Fluo} *\left( {1 - e^{{ - \frac{t}{{\tau_{Fluo} }}}} } \right)$. where C_Fluo_ and τ_Fluo_ were respectively related to the fluorescence plateau value and to the fluorescence time constant, and

$$ \Delta_{THz} { }\left( {\text{t}} \right) = 100 - { }C_{THz} *\left( {1 - e^{{ - \frac{t}{{\tau_{THz} }}}} } \right) $$

where C_THz_ and τ_THz_ were related to the THz-ATR plateau value and to the THz-ATR time constant, respectively. C_Fluo_ and τ_Fluo_ were compared respectively to C_THz_ and τ_THz_ in order to perform quantitative and qualitative comparisons between the fluorescence and the THz-ATR modalities.

## Results

### Raman signature of live cells exposed to µsPEF

**Figure 1:**
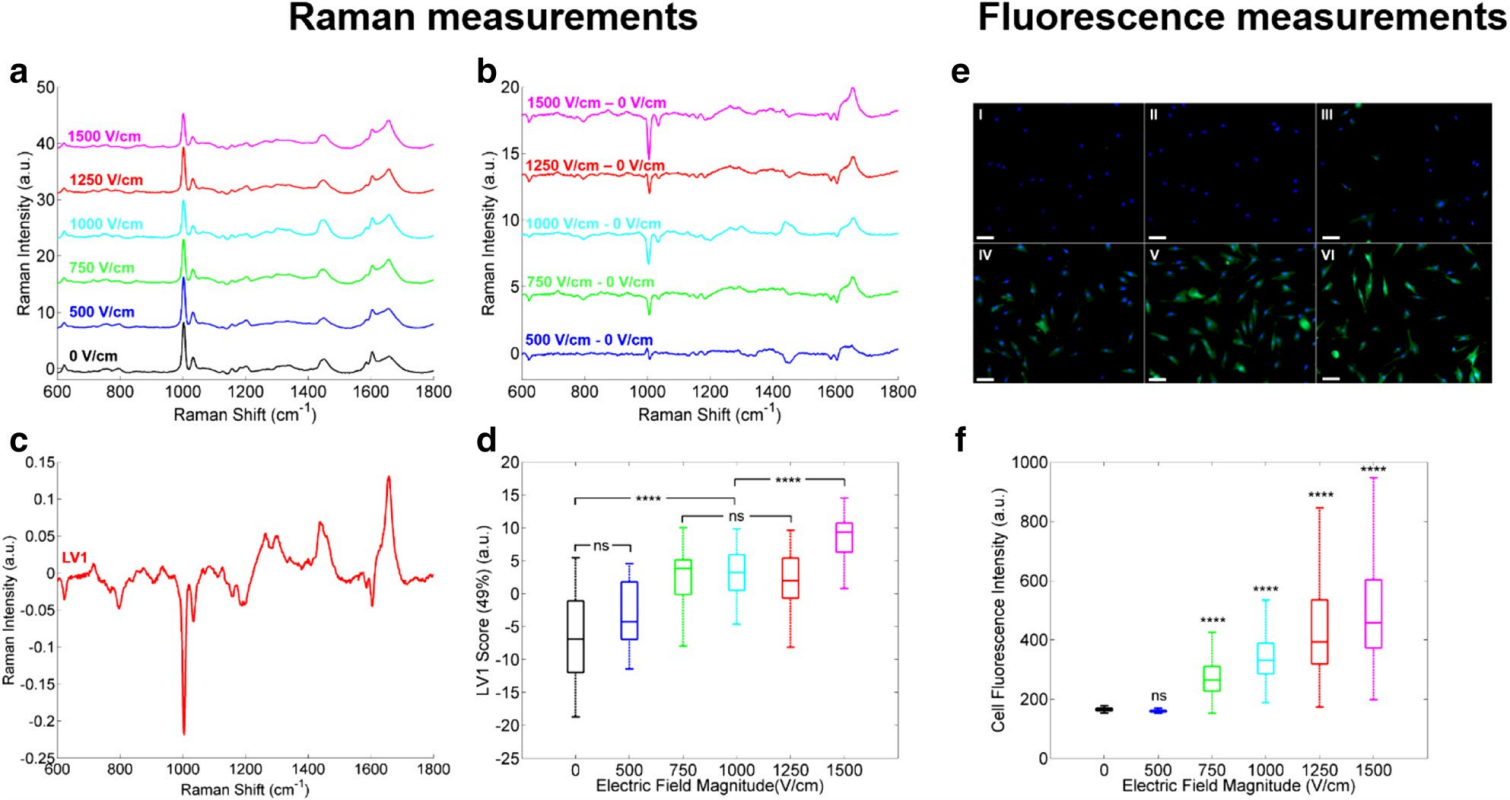
Raman (**a**–**d**) and fluorescence (**e**–**f**) measurements of live haMSC exposed to µsPEF. The magnitude of the electric fields was 0 V/cm (black), 500 V/cm (dark blue), 750 V/cm (green), 1000 V/cm (light blue), 1250 V/cm (red) or 1500 V/cm (magenta) The other pulse parameters were fixed to 8 pulses, 100 μs and 1 Hz. (**a**) Mean normalized Raman signature of each group of cells in arbitrary units (a.u.). (**b**) Mean normalized Raman signature of each group of cells minus the mean normalized Raman signature of cells under sham exposure (0 V/cm). (**c**) Loading of LV1 from PLS analysis. (**d**) The LV1 score of each group. The percentage of variance supported by the LV1 is indicated in brackets. Three independent experiments were performed at least for each group. (**e**) Representative examples of Hoechst 3342 (blue) and Yo-Pro-1 (green) fluorescence images of haMSC exposed to µsPEF of 0 V/cm (I), 500 V/cm (II), 750 V/cm (III), 1000 V/cm (IV), 1250 V/cm (V) and 1500 V/cm (VI) magnitude. Scale bar = 100 μm. (**f**) Quantitative analysis of the Yo-Pro-1 fluorescence intensity of haMSC exposed to µsPEF. Three independent experiments were performed at least for each group. &’’_Student’s t-test: ns (non-statistically significant): *p* value > 5%, ****p value ≤ 0.01%.

The Raman signatures of live haMSC cells exposed to µsPEF under different field magnitudes were acquired on the cytoplasm using a confocal Raman microscope. In Fig. 1a the raw spectra for the different conditions assayed are shown. To enhance the spectral modifications associated with the µsPEF delivery, the difference between the mean normalized Raman spectra with respect to those of the control group (i.e. 0 V/cm) was calculated for each electric field intensity. (Fig. 1b). Confirming our previous studies^21,22^, several Raman vibrations were affected by the delivery of the electric pulses. The phenylalanine ring breathing vibrational mode at 1003 cm^−1^ and the Amide I band at 1658 cm^−1^ were the predominant peaks. A dose effect was noticed when monitoring the intensity of these two peaks. The decreasing of Raman peaks at 1033 and 1605 cm^−1^ with increasing field magnitudes, confirmed the strong effect of the µsPEF on the phenylalanine residues. In addition, the multivariable analysis by Partial Least Square regression (PLS)^39^ permitted to reinforce these dose effect results. The example of the first Latent Variable (LV1) loading depicted in Fig. 1c shows how the major Raman peaks contributing to the LV1 appeared clearly at 1003 and 1658 cm^−1^. The 1448 cm^−1^ stretching mode of CH vibration, mainly attributed to lipids^40^, was also part of the LV1 which is consistent with the known effect of the µsPEF on the lipid cell bilayer^11^. Statistical analysis of the LV1 scores (Fig. 1d) led to distinguish three groups with strong significant intergroup differences and no statistically significant intragroup difference: (0, 500 V/cm), (750, 1000 and 1250 V/cm) and 1500 V/cm. The uptake of the non-permeant fluorescent dye YO-PRO-1 was measured to quantify the permeabilization of haMSC cells exposed to µsPEF under the exact same conditions as in the Raman experiments. Figure 1e shows representative examples of fluorescence images acquired for the different electric field magnitudes delivered to the cells. The quantification of YO-PRO-1 fluorescence intensity into the cells shown in Fig. 1f presented a linear response as a function of the field magnitude. For field magnitudes above 500 V/cm, the YO-PRO-1 cell fluorescence intensity was significantly higher than in the control group and increased with the magnitude of the electric field applied. The high standard deviation of the fluorescence intensity measurements might be associated with differences in the cell shape, the cell orientation or even in a possible shielding effect produced by the adjacent cells^41^.

**Figure 2:**
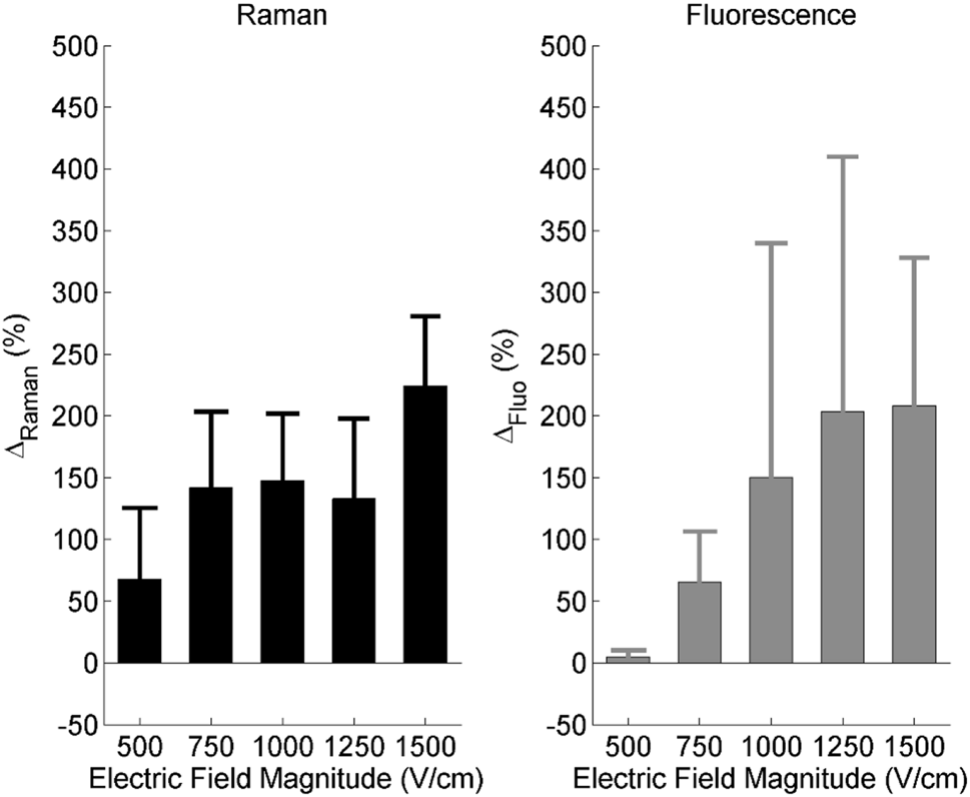
Quantitative comparison of Raman and fluorescence modalities in the case of live haMSC exposed to different electric field magnitudes under exactly the same experimental conditions. Δ_Raman_ and Δ_Fluo_ respectively represent the normalized relative Raman (left) and fluorescence (right) signals with respect to the corresponding control groups. The electric field magnitude varied from 500 to 1500 V/cm. The other pulse parameters were fixed to 8 pulses, 100 μs and 1 Hz for all the experiments. The bar and the error bar represent the mean and the standard deviation of the distribution, respectively, per experimental condition (n ≥ 3).

In order to compare the Raman data with the fluorescence measurements, the normalized relative Raman and fluorescence signals, respectively symbolized by ΔRaman and ΔFluo, were calculated for the different magnitudes of the delivered electric field (Fig. 2). At 500 and 750 V/cm, Raman modality provides higher difference to the control group than fluorescence modality. In particular, no difference in the fluorescence signal was noticed between the 500 V/cm group and the control group, whereas an important 68% relative increase of the Raman signal was recorded at 500 V/cm. When the cells were exposed to 1000 V/cm, the relative evolution of the signal was basically the same for the two modalities. At 1250, the fluorescence modality displayed an increase in the relative signal magnitude of around 200%, while the Raman modality did not. At the highest exposure condition assayed (1500 V/cm), the Raman relative change increased to more than 200%, while for fluorescence the relative change remained around 200%, a value similar to the one observed at 1250 V/cm. It is important to remind that the fluorescence results are highly dependent on the fluorescent dye used in the experiments, its size, its charge, its external concentration, and, when it applies, its binding conditions. On the contrary, CRMS is a label-free optical technique and thus the results are related to the intrinsic chemical composition of cells.

### Terahertz dynamics of non-permeant molecule efflux from live cells after µsPEF

**Figure 3:**
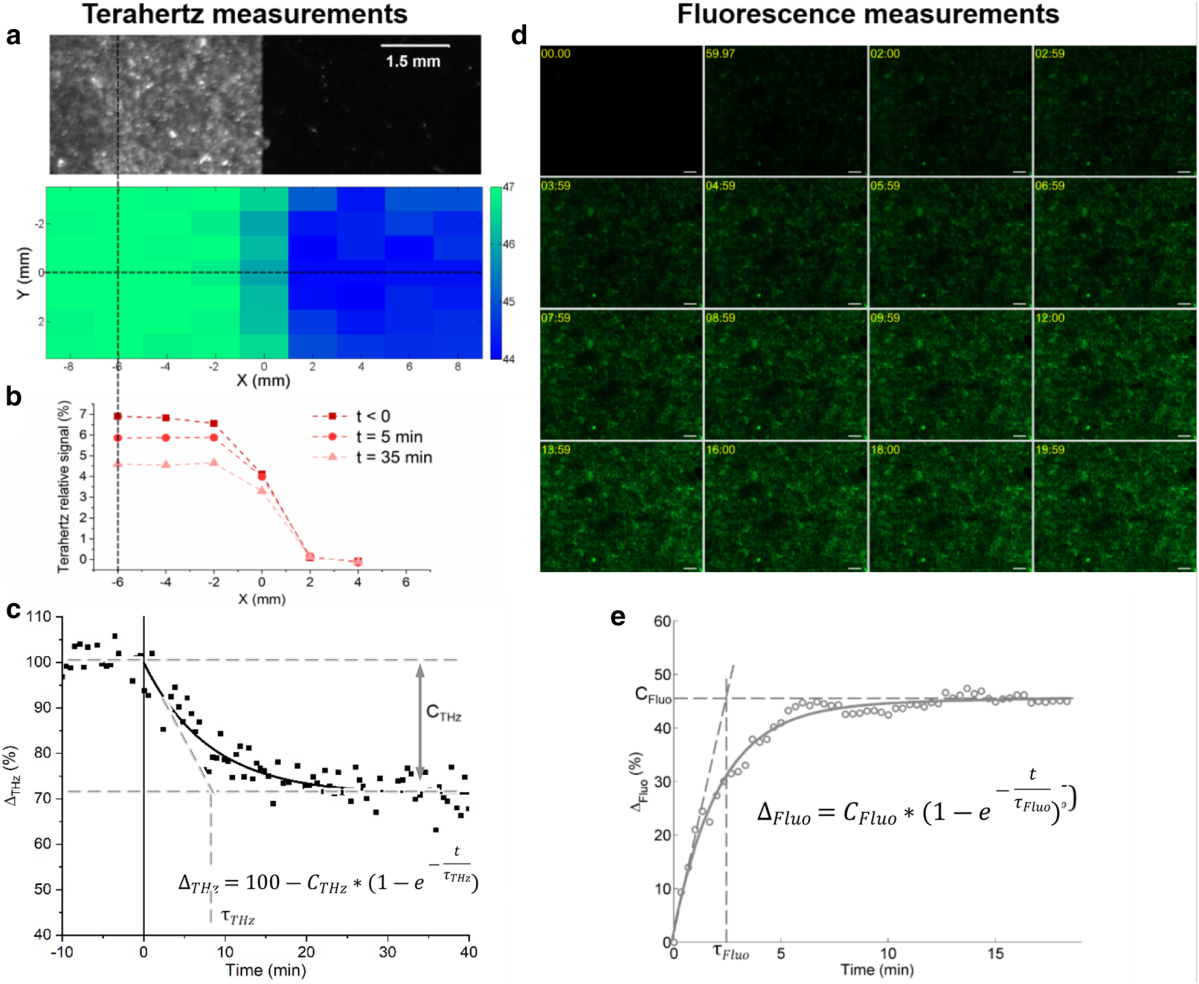
Illustration of the THz-ATR (**a**–**c**) and fluorescence (**d**–**e**) measurements. (**a**) Bright-field (top) and THz-ATR (bottom) images of areas covered (left) or not (right) with the MDCK epithelial cell monolayer. (**b**) Representative examples of the THz-ATR relative signal of a sample along the X axis (dotted horizontal line in **a**) at three times: before, 5 and 35 min after the delivery of the electric pulses. (**c**) Example of the time-evolution of the normalized THz-ATR relative signal of live MDCK cells exposed to µsPEF. Square corresponds to the measurements and the black line to the fitted mathematical function reported in the same panel. (**d**) Representative example of YO-PRO-1 fluorescence time-lapse images of live MDCK cells exposed to µsPEF. The delivery of the electric pulses was at time 00:00. For each image, the relative acquisition time was reported. Scale bars = 100 μm. (**e**) Example of the time-evolution of the normalized YO-PRO-1 fluorescence relative signal of live MDCK cells exposed to µsPEF. Circle corresponds to the measurements and the grey line to the fitted mathematical function reported in the same panel. For all these graphs, the parameters of the µsPEF were 8 pulses, 1250 V/cm, 100 μs and 1 Hz.

A real-time Terahertz-Attenuated Total Reflection experimental setup is used to assess the dynamics of µsPEF and live cells interaction, for the first time. The THz-ATR signals of MDCK cells were recorded before and during 40 min after the delivery of the electric pulses and allowed to analyze the dynamics of the membrane permeabilization. In Fig. 3a, a bright field picture and the corresponding THz-ATR signal are shown for a typical sample (an area with cells on the left, with about 2000 cells per pixel, versus an area without cells on the right). These images show an approximate 7% peak amplitude signal difference between cells and their outer medium, further called THz-ATR relative signal. In the supplementary information and reference^31^ we bring the demonstration that the THz-ATR signal can be used as a label-free biomarker of the intracellular concentration of a large range of molecules, from small metabolites to peptides and proteins, and used as a non-invasive quantitative measurement of cell permeabilization. Figure 3c displays the evolution of the normalized THz-ATR signal after the delivery of the electric pulses and the mathematical exponential function used to fit the measurements. The relative THz-ATR signal decrease observed after the delivery of the electric pulses suggests a change in the composition of the cytosolic content of cells associated to the leakage of molecules from the cells due to their electropermeabilization. A possible concomitant osmotic water uptake into cells could also decrease the molecules concentrations in the cytosol and hence the THz-ATR signal. It was for instance observed in lymphoblasts exposed to nsPEF^42^ with a characteristic time shorter than 1 min. On the contrary, no swelling was observed in adrenal chromaffin cells^43^ exposed to nsPEF. In our THz-ATR experiments (Fig. 3c), we did not observe rapid variations of the signal immediately after the µsPEF delivery, compared to the signal before µsPEF, within the experimental precision. The signal is dominated by slower dynamics than the one observed in lymphoblasts, so we consider that water uptake plays a minor role in MDCK cells under the µsPEF parameters applied in our study. Figure 3d shows an example of the time-lapse fluorescence microscopy images of MDCK cells exposed to the same electric pulse conditions. The uptake of YO-PRO-1 into cells was assessed in order to quantify and compare the dynamics of the electropermeabilization process. A representative example of the evolution of the normalized relative fluorescence signal and the associated mathematical exponential function are shown in Fig. 3e.

**Figure 4:**
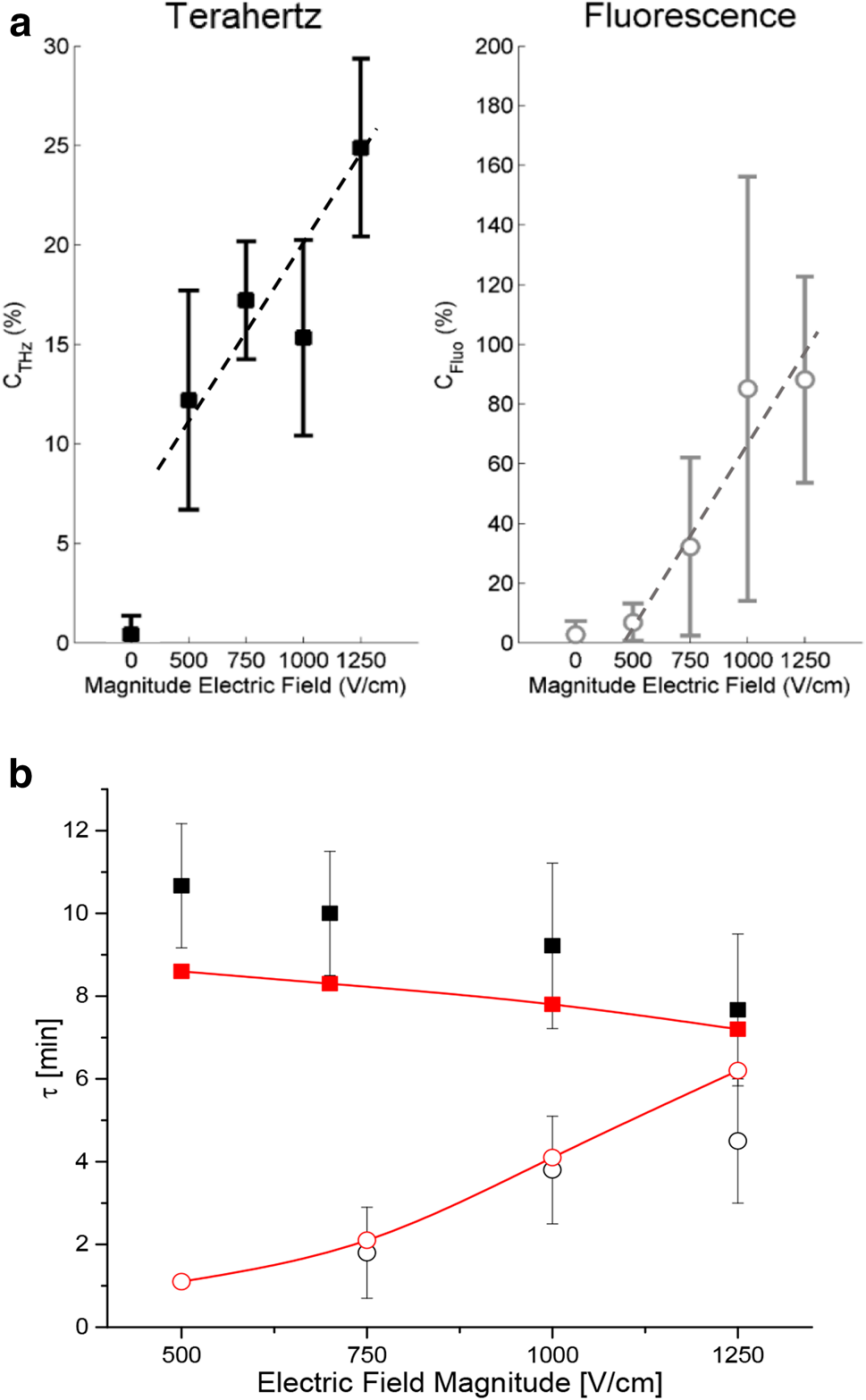
Evolution of the exponential function parameters fitting the THz-ATR and fluorescence measurements of live MDCK cells exposed to electric pulses with different electric field magnitudes. (**a**) Plateau values C_THz_ and C_fluo_ for the THz-ATR and YO-PRO-1 fluorescence normalized signals. Dashed lines are guides for the eye. (**b**) Time constants of the THz-ATR (squares) and YO-PRO-1 fluorescence (circles) normalized signals calculated from the experimental data (black) or from the theoretical model (red). The error bar represents the standard deviation of the data set for each experimental condition (n ≥ 3). τ was not determined if the coefficient of determination (R^2^) between the model and the measurement was below 90%. The electric pulse parameters were fixed to 8 pulses, 100 μs and 1 Hz.

In both THz-ATR and fluorescence microscopy modalities, the measurements were fitted to exponential functions. Two quantitative parameters, a plateau value (C_THz_ and C_Fluo_, respectively) and a time constant (τ_THz_ and τ_Fluo_) can be extracted from these functions, for the various field amplitudes applied. Both measurements refer to signal average values divided by the number of cells: then the normalized signals can directly be compared. First, Fig. 4a shows the dependence of the plateau values on the electric field intensities for both techniques. This parameter gives information about the total relative change accumulated after the electric pulses delivery. The plateau value relative change is much lower in percentage in the case of the THz-ATR than in the case of the fluorescence except at the lowest field amplitude tested. Indeed, interestingly, at 500 V/cm, the fluorescence plateau value remained close to 0, indicating that no change was detected under such conditions whereas the THz-ATR plateau value was around 12 to 13%. This would indicate that the detection threshold of THz-ATR setup was lower than the detection threshold of fluorescence. This would indicate that the detection threshold using the THz setup is lower than the detection threshold using YO-PRO-1 fluorescence. In fluorescence measurements, the signal depends on a single type of molecule, the YO-PRO-1 probe (molar mass 629 Da). In THz-ATR measurements, the signal depends on a wide range of molecules, including molecules much smaller than the YO-PRO-1 (such as sugars, as well as all the amino acids). Therefore, if the membrane is not permeant to molecules as large as YO-PRO-1 for weak amplitude µsPEF, no dynamics can be followed by fluorescence. On the contrary, dynamics can be measured by THz-ATR since smaller molecules can cross the membrane and contribute to the generation of a THz-ATR signal. When increasing the electric field magnitude, a dose–response effect of the THz-ATR and fluorescence plateau values was noticed (Fig. 4a). At the two highest electric field intensities studied (1000 and 1250 V/cm) it can be observed that C_THz_ displays a considerable increase while C_Fluo_ is almost constant for both field intensities. Figure 4b shows the values of the exponential functions time constants obtained for both techniques after fitting the measurements as well as the corresponding values obtained from the theoretical model developed and described in full detail in the supplementary information. The agreement between experimental data and model is good considering that the model parameters are the same for both fluorescence and terahertz values. As observed, there is a clear difference in the behavior of this parameter with the electric field intensity between the two techniques. While the THz-ATR time constant decreases with the field magnitude, the fluorescence time constant increases with the field intensity. This observation is discussed in detail in the following section.

## Discussion

In this study, we report a qualitative and quantitative comparison between fluorescence microscopy and two label-free optical methods, CRMS microscopy and THz-ATR measurements, to investigate the effects of µsPEF on live cells. We demonstrated in a previous study that the CRMS results originate from the effect of electric pulses on amino-acids and proteins present in live cells via the modification of phenylalanine and Amide I vibrational modes^21^. Additionally, a numerical model supported our results by demonstrating the effect of electric pulses on the folding state of membrane proteins^44^. In the present study, a dose–response effect was noticed in the Raman signature of live cells when increasing the field magnitude. We may attribute the three groups (first: 0 and 500 V/cm; second: 750, 1000 and 1250 V/cm; third: 1500 V/cm), identified by statistical analysis, to three different states of the plasma membrane, meaning no permeabilization, reversible permeabilization and irreversible permeabilization. Therefore, in this manuscript, using a PLS approach, we further extend the analysis of the data reported in our previous manuscript^22^ where the multivariate analysis was performed using the Principal Component Analysis (PCA) method, where a principal component is a spectrum supporting a specific variance of the data set. Whether we use PLS or PCA, 0 and 500 V/cm result in no significant change with respect to the unpulsed samples, and we can consider that no permeabilization of the membrane is detectable using CRMS. On the opposite, the differences between reversible (at 1000 V/cm) and irreversible permeabilization (at 1500 V/cm) could not be detected by the PCA approach in our previous study and are now detected using the PLS approach (Fig. 1). The new analysis (using PLS) does not contradict our previous analysis (using PCA). Both PCA and PLS are valid, strong and complementary. PCA is based on an unsupervised analysis of the variance, meaning that PCA is “blind”, no external observation variable being considered in this case. Therefore, PCA is important to demonstrate that there is actually an effect of the treatment (comparison between controls/shams and treated groups) and for this reason we used it previously. For this reason, as well, no significant dosage effect was reported in reference^45^. On the contrary, PLS analysis is supervised: an external observation variable is known at the time of the analysis (e.g. the pulsed field amplitude). Each spectrum of the data set was thus attributed to the pulse amplitude at which the spectra were collected. Then, if there is a dosage effect as a function of the pulse amplitude, we should find it. Actually, we had to use PLS for the comparison of CRMS data and fluorescence data here reported because in the analysis of the fluorescence data we have to know the correspondence between fluorescence amplitude and pulse amplitude. For all these reasons, we found here a significant dosage effect. Thus, the Raman signature of cells would be a biomarker of the permeabilized state of the plasma membrane. It must be noticed that fluorescence microscopy results depend on the properties (size, charge, etc.) of the fluorescence dye used^46,47^. The results would be different with another fluorescence dye such as calcium green, Fluo-4, etc. Fluorescence yield of YO-PRO-1 is also sensitive to its binding to various cell binding sites, making it a semi-quantitative technique. On the contrary, CRMS is label free and quantitative.

Thanks to the THz-ATR measurements, the leakage of cytosolic molecules from cells exposed to µsPEF was thoroughly investigated. Even though amino acids and proteins leakage was extensively studied more than 50 years ago when the process of electroporation was termed “membrane dielectric breakdown”^48^, this is not usually evoked when the consequences of the cell membrane permeabilization are listed. However, like most of the non-permeant molecules, amino acids and proteins will also cross the cell membrane similarly to the well-known leakage of other internal substances like the ATP^49^. As expected, a dose–response effect was observed: an increase in the electric field magnitude was associated with a higher decrease of the normalized THz-ATR relative signal. This relation can be attributed to the idea that at higher electric fields, not only the number, but also the size of membrane pores is increased. Some studies performed with fluorescence microscopy have shown that the permeabilization detection threshold depends on the fluorescence dye size^33,47^ while other molecular properties such as the charge can also impact the results^46^. Thus, low electric fields allow only the small molecules to cross the plasma membrane, while high electric fields allow small and larger molecules to cross the plasma membrane. As the THz-ATR signal originates from the presence of small (~ 100 Da) to large (~ 200 kDa) molecules in large concentrations inside the cells (see Supplementary Information), at low electric fields the THz-ATR signal variations will be dominated by the contribution of small molecules such as amino acids. Increasing the electric field, will allow the crossing of bigger molecules and accordingly a shift of the mean molecular weight towards higher values. Then, the THz-ATR signal will be dominated by the contribution of the larger molecules, which will diffuse from the cells more slowly than the small molecules. Summarizing, the THz-ATR signal represents a global response of the leakage of molecules of different sizes depending on the applied electric field intensity. On the opposite side, the fluorescence microscopy technique used in this study provides information only about the dynamics of a single molecule with a fixed size (~ 630 Da for YO-PRO-1), regardless the intensity of electric field applied.

In this scenario it is therefore possible to justify why the time constants, obtained after fitting an exponential function to both the fluorescence and THz-ATR signal variations, have different behaviors. This can be explained using the transient permeabilization model presented in the Supplementary Information part. Our model is based on Fick’s first law and the solubility-diffusion model, as well as on the assumption that the electropore size exponentially reduces with time after the pores creation by µsPEF^50^ whatever the structure of this electropore. The evolution of the molecule population crossing the permeabilized cell membrane is given by a time-dependent permeabilization which depends on an effective diffusion-area fraction $$K$$ (which is variable) and on the cytosol diffusion constant $$D_{c}$$ of the various types of molecules. This model shows that the time constants for terahertz and fluorescence mostly depend on $$K$$ and decrease with the ratio $$r_{s} /r_{p}$$, where $$r_{s}$$ and $$r_{p}$$ are the solute and pore radius, respectively (see Supplementary Information, Fig. S3). Therefore, the behavior of $$\tau_{Fluo}$$ and $$\tau_{THz}$$ versus the electric field $$E$$ can be explained as follows. On the one hand, for YO-PRO-1 fluorescence measurements, the increase of $$E$$ leads to an increase of $$r_{p}$$ while $$r_{s}$$ does not change, therefore to a decrease of $$r_{s} /r_{p}$$ and thus to an increase of $$\tau_{Fluo}$$ versus $$E$$. On the other hand, for $$\tau_{THz}$$ a more complex reasoning must be done. Because THz-ATR signal encompasses a wide range of molecule sizes, thus of $$r_{s}$$, and because $$ r_{p}$$ increases with the value of the electric field $$E$$, the evolution of $$\tau$$ versus $$E$$ is then a complex trade-off between the variations of both $$r_{p}$$ and $$r_{s}$$. For example, for molecules smaller than YO-PRO-1 (of a given $$r_{{s\left( {THz} \right)}}$$ which will be smaller than the $$r_{{s\left( {Fluo} \right)}}$$ of the YO-PRO-1), $$r_{{s\left( {THz} \right)}} /r_{p}$$ will be smaller than $$r_{{s\left( {Fluo} \right)}} /r_{p}$$ which explains both that $$\tau_{THz}$$ is larger than $$\tau_{Fluo} $$ and that permeabilization by THz-ATR is already detected at 500 V/cm while not yet detected by fluorescence. The simulations displayed in Fig. S4 show that, with increasing $$E$$, there is an overall increase of $$\tau_{Fluo}$$ (using YO-PRO-1) as well as higher and decreasing $$\tau_{THz}$$, as experimentally observed in Fig. 4b.

Finally, it must be mentioned that the SNR was higher in the case of the fluorescence experiments than in the THz-ATR ones (Fig. 3c, e). This might be associated with the technology maturity. While fluorescence microscopy is a well-established technology, THz-ATR technology is still under development.

## Conclusions

In conclusion, two different label-free optical methods (CRMS microscopy and THz-ATR measurements) investigated the strong impact of cell permeabilizing µsPEF on the chemical composition of live cells. THz-ATR measurements were performed during electroporation and used to monitor in real time the changes in composition of the cytosol of electropermeabilized cells. Finally, a model describes the dynamics of non-permeant molecule efflux from live cells after µsPEF based on terahertz data.

The Raman and terahertz modalities were quantitatively and qualitatively compared to fluorescence microscopy. Table 1 summarizes the terahertz, Raman and fluorescence modalities in the framework of the interaction between µsPEF and live cells. Table 1, with the strengths and weaknesses of the three compared techniques, will allow the reader to decide which technique should be used depending on the scientific goals of a particular experiment. The present paper will allow the reader to gain access to other modalities of label-free microscopy that could be used when fluorescent dyes cannot be used or when other information different from the influx of a molecule to the cytoplasm is interesting for the experiment. Moreover, the complementary information given by the different optical methods gives a more complete description of the phenomena associated to cell electroporation and their dynamics.

**Table 1:** Main characteristics, strengths and weaknesses of the three technologies compared.

	Raman	Fluorescence	Terahertz
Origin of the signal	Intrinsic chemical composition of the cell	Amount of fluorescent dye inside the cell	Amount of diverse metabolites and proteins inside the cell
Signal acquired	Vibrational spectrum	Fluorescence intensity	THz peak magnitude
Origin of the signal evolution after the delivery of µsPEF	Changes in the molecular composition of the cell	Internalization of non-permeant fluorescence dye into the cell	Leakage of molecules across the membrane
Detection threshold (V/cm) in comparison with control group	≤ 500	> 500 and ≤ 750	≤ 500
Dose effect	Signal maybe related to the permeabilization state of the plasma membrane	Signal increases with the electric field magnitude for electric field magnitude above the detection threshold	Signal increases with the electric field magnitude
Label	No	Yes	No
Time resolution	Very low (~ 60 s)	Good (~ 0.3 s)	Low (~ 10 s)
Spatial resolution	Very good (~ 1 μm)	Very good (~ 1 μm)	Very low (~ 2500 μm)
Requirements for signal quantification	Normalization of the spectrum	Internal references necessary	Internal reference necessary
Signal stability	Excellent (~ hours)	Low (~ 10 s) (photobleaching)	Excellent (~ hours)
Data processing	Multivariate analysis	Univariate analysis	Univariate analysis
Sample preparation	Specific substrate and solution	Labeling protocol	Specific substrate and solution
Technology maturity	Research set-up	Commercialized equipment	Research set-up

## Supplementary information

Supplementary file
