# Supplementary material for: Monitoring the molecular composition of live cells exposed to electric pulses via label-free optical methods
Source: Sci Rep. 2020 Jun 26;10:10471. doi: 10.1038/s41598-020-67402-x (PMC7319994; doi:10.1038/s41598-020-67402-x)
Supplement: Supplementary file 1 — Supplementary file [file 41598_2020_67402_MOESM1_ESM.pdf]

Monitoring the Molecular Composition of Live Cells Exposed to Electric Pulses via Label-free Optical Methods

Antoine Azan, Marianne Grognot, Tomás García-Sánchez, Lucie Descamps, Valérie Untereiner, Olivier Piot, Guilhem Gallot, and Lluis M. Mir

SUPPLEMENTARY INFORMATION

Origin of the terahertz signal

The recorded terahertz signal variations originate from changes of the cytosol molecules concentration. More precisely, the THz relative signal difference between the cells and their outer medium is proportional to the mass concentration of all intracellular molecules, from ions, metabolites to proteins. To demonstrate this, we measured the THz relative signal obtained between simple solutions of molecules compared to pure water. Acquisitions of the THz relative signal were made on amino-acids, peptides and proteins up to 250 kDa. As shown in **Figure S1a** for a few examples, there was a linear relationship between a molecule mass concentration (in the biological range) and the THz relative signal generated. These slopes values are the THz relative signal sensitivity to mass concentration for each molecule. Values of all THz relative signal sensitivity given the size of the molecules tested are given in (in **Figure S1b**). The observed evolution of THz sensitivity with molecular weight can be understood and fitted by a simple volume model, as detailed in [1].

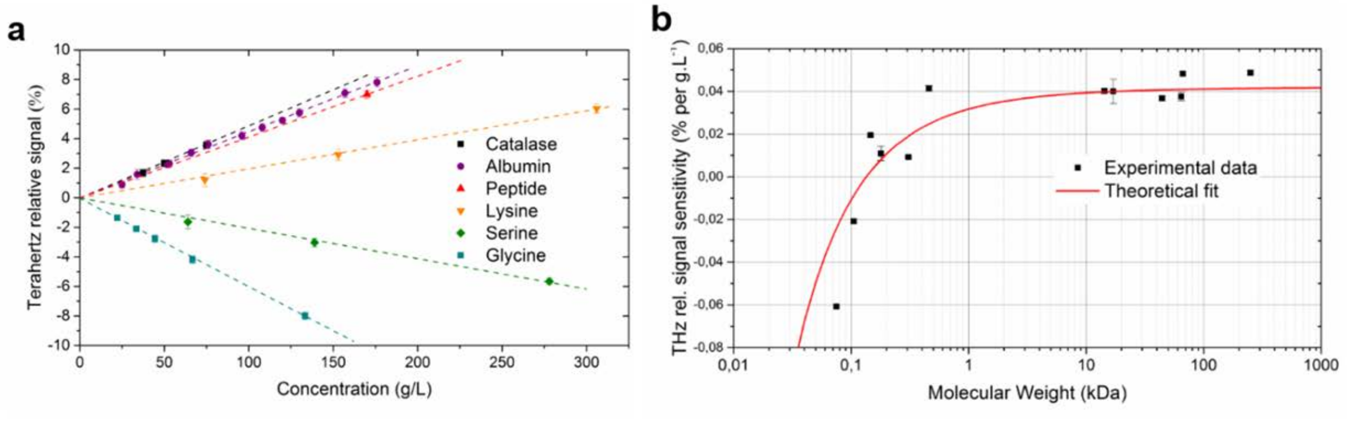

***Figure S1**: THz relative signal for amino-acid, peptide or protein solutions shows a linear relationship with mass concentration (a). Each slope illustrates the THz relative signal sensitivity to mass concentration of a given molecule. When plotted with respect to the molecular weight (b, black squares), sensitivity shows a global tendency that can be well fitted by a theoretical model (b, red line) such as described in [1]. From the smallest amino-acid to molecules of 1 kDa, the signal sensitivity sharply increases, meaning that a THz measurement will reflect both concentration and molecular weight of very small metabolites in a complex fashion, biased towards higher molecular weight for a given molar concentration. Above 2 kDa, sensitivity reaches a constant value (within a 12% margin), meaning that a THz measurement will then only probe the protein mass concentration regardless of their molecular weight.*

Transient permeabilization model

The cytoplasmic membrane is a biological barrier that separates the interior of the cell to the extracellular space. It controls the flux of molecules in and out of the cells, being selectively permeable to specific molecules. In electropermeabilization, the membrane permeability can be transiently increased by applying an electric field, so that non-permeant molecules can cross the membrane.

We develop here a model to explain the behaviour of τ_{THz}, which reflects the characteristic time for the efflux of molecules from the cytosol through the transiently permeabilized membrane. We report here how this transient permeability model applies to molecules efflux from the cells inside or to molecules influx depending on the initial concentration conditions.

To model this transient permeabilization, we consider here a membrane of surface A surrounding a cell of volume V, and the net efflux j of molecules of molecular weight M through the membrane, given by Fick's first law and the solubility-diffusion model for permeability [2], as

$$j = -KD_c \frac{\Delta C}{\delta}, \tag{1}$$

where D_c is the diffusion constant in cytosol, δ the membrane thickness, $C = N/V$ the concentration inside the cell and K the effective diffusion area fraction, (that is the fraction of the unit area through which diffusion is effective for a molecule of mass M and Stokes radius r_S). The effective diffusion area fraction K varies from 1 (fully open membrane) to 0 (closed membrane) and it is usually several orders of magnitude smaller than 1. The variation of the number of molecules N inside the cell is then

$$\frac{dN}{dt} = -\gamma N \qquad \text{where } \gamma(t) = \frac{A}{V}\frac{KD_c}{\delta} \tag{2}$$

is the time-varying transfer rate constant. Let the permeability $p = KD_c/\delta$, the transfer rate writes $\gamma = \alpha p$ where $\alpha = A/V$ is a constant depending only on the geometry of the cell. For efflux from the cytosol, the number of molecules N_e is given by

$$N_e(t) = N_0 \exp\left[-\int_0^t \gamma(u)\, du\right], \tag{3}$$

where $N_0 = N(t = 0)$ is the internal concentration in the unpermeabilized cell. On an equivalent basis, for influx of fluorescent molecules into the cell, the solution N_F is given by

$$N_F(t) = C_F\, V \left[1 - \exp\left(-\int_0^t \gamma(u)\, du\right)\right], \tag{4}$$

where C_F is the concentration of fluorescent molecules in the extracellular space, assuming an infinite reservoir of molecules.

An applied electric field E transiently modifies the effective diffusion area fraction $K(t)$, which depends on the size of the electropores r_p and on the Stokes radius of the molecules r_s, following a Renkin model for diffusion through pores (see Figure S2) [3]. In mammalian cells, r_p is found to decrease exponentially after electropermeabilization with a time constant τ_R independent of E [4], $r_p(t) = r_{p0}(E)\, e^{-t/\tau_R}$. We then model the evolution of the molecules through the cytosol membrane, integrating Eqs. (3) and (4), using

$$\gamma(t) = \alpha \frac{K(t, r_{p0}, r_s, \tau_R)\, D_c(r_s)}{\delta}. \tag{5}$$

The diffusion is given by Stokes-Einstein equation,

$$D_c = \frac{k_B T}{6\pi\eta r_s} \quad \text{with} \quad r_s = \left(\frac{3M}{4\pi\rho N_A}\right)^{1/3}$$

where η is the viscosity of the cytosol, ρ the solute density, close to 1.37 g/cm^3 for a wide range of solute molecular weight [5] and N_A the Avogadro's number. Additional parameters can be found in Table 1.

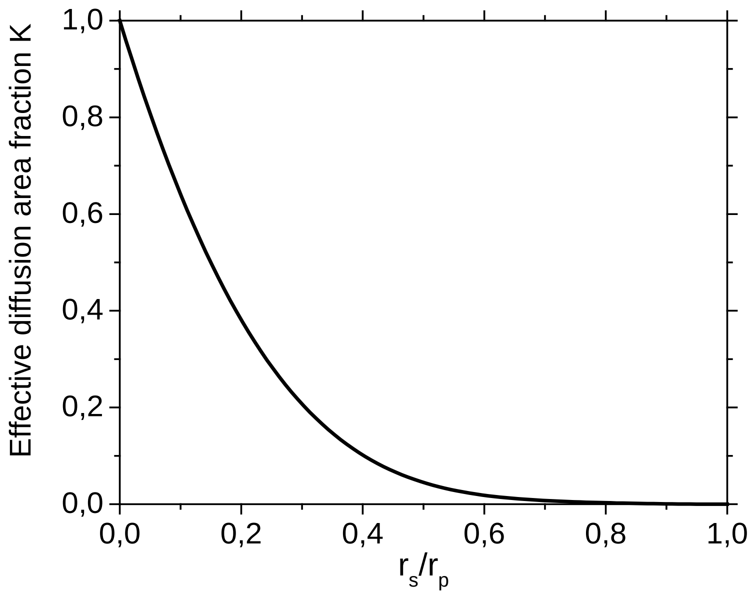

Figure S2 : Effective diffusion area fraction K versus r_s/r_p.

The evolution of $N_e(t)$ and $N_F(t)$ are close to an exponential, and can be described by a characteristic decay time τ. Since γ depends on K and D_c, which both depend on r_p and r_s, we investigated the relative contributions of K and D_c on τ. We found (see **Figure S3**) that τ depends almost entirely on the K contribution, an increase of the ratio r_s/r_p contributing to a decrease of τ, and very little on D_c.

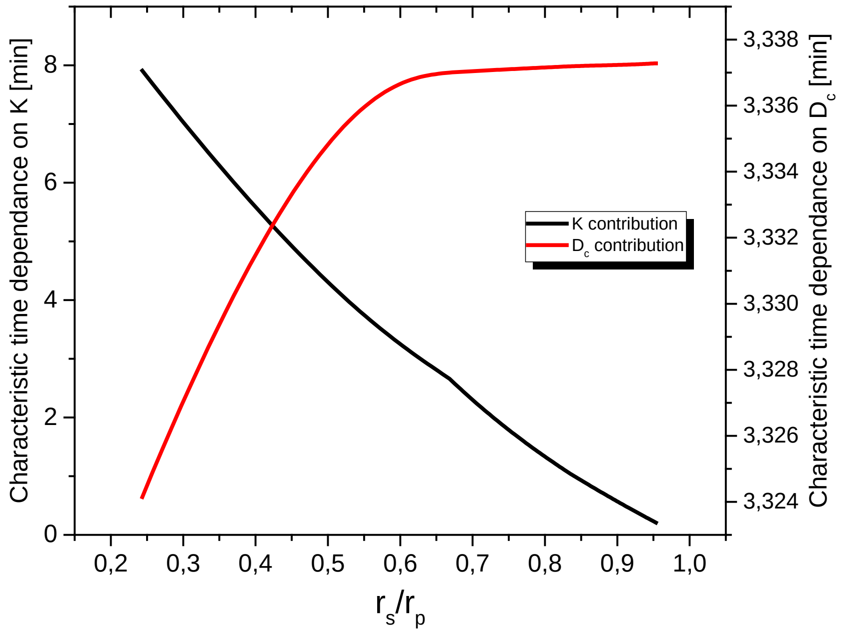

Figure S3: *Relative contributions of K (left) and D_c (right) (the other one remaining constant) on the characteristic time τ, versus r_s/r_p.*

It is then possible to predict the evolution of τ with respect to the molecular weight M of the crossing molecules through the cell membrane. Let consider a decrease of M with a constant electric field so that r_p is constant. The effect of D_c is negligible from **Figure S3**, and the contribution relative to K leads to an increase of τ. If now we increase E for the same class of molecules (YO-PRO-1 for instance), then r_p increases thus r_s/r_p decreases, so that τ increases with respect to E. At last, in the case of the terahertz signal, the recorded signal average the contribution of a large set of molecules of different sizes. An increase of E then shifts the molecular mass of the probed molecules towards higher values, amplified by the increased sensitivity of the terahertz measurement to higher molecular mass in the 100-2,000 Da region (see Figure S1). The evolution of τ versus E is then a complex trade-off between the increase of both r_p and r_s. The simulations shown in **Figure S4** exhibit an increase of the characteristic time if the transport of a single molecule size is considered alone. Observe, for example, the line corresponding to a molecular weight of 629 Da (corresponding to YO-PRO-1). On the contrary, if the calculation of the time constant is performed considering various molecular sizes crossing the membrane together, depending on the electric field applied and thus the pore radius, a decreasing terahertz time constant is obtained with increasing electric field in agreement with the experimental observations.

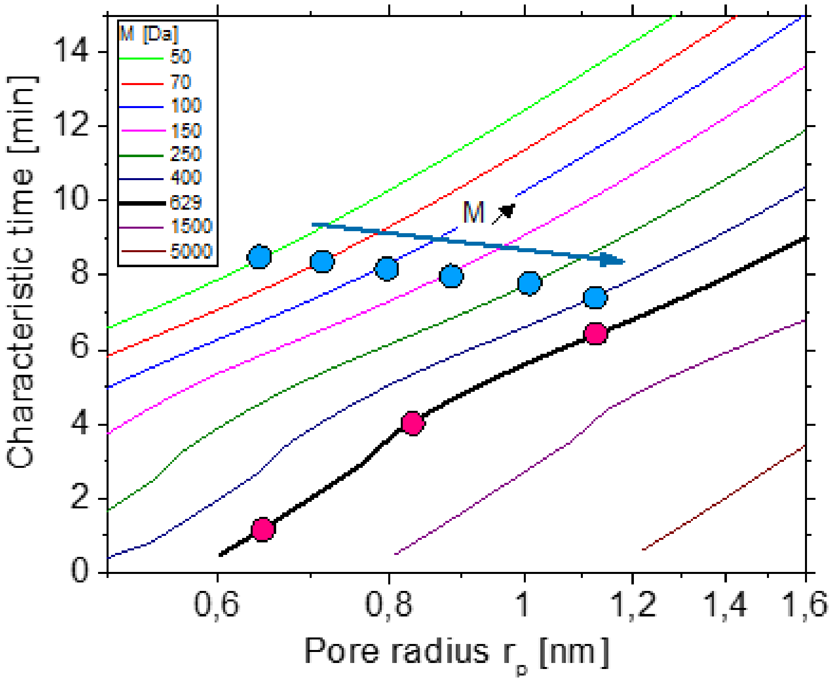

Figure S4: *Evolution of the characteristic time τ versus pore radius r_p, for YO-PRO-1 fluorescence (red) and terahertz (blue) signals, for several molecules of molecular mass M (YO-PRO-1 mass is 629 Da).*

Table 1: *Other model parameters.*

Parameter	Value
Solute density ρ	1.37 g/cm^3 [5]
YO-PRO-1 molar mass	629 Da [6]
YO-PRO-1 Stokes radius	0.53 nm [6]
Cell radius	10 µm
Protein concentration	165 g/L [7]
Metabolites concentration	9 g/L [7]
Ion concentration	3 g/L [7]
Viscosity µ	0.11 Pa.s [8]

REFERENCES:

[1] M. Grognot and G. Gallot, "Relative Contributions of Core Protein and Solvation Shell in the Terahertz Dielectric Properties of Protein Solutions," *J. Phys. Chem. B,* vol. 121, pp. 9508-9512, 2017.

[2] J. Crank, *The mathematics of diffusion. Second Edition.* : Oxford Univ. Press, 1975.

[3] E. M. Renkin, "Filtration, diffusion, and molecular sieving through porous cellulose membranes.," *J. Gen. Phys.,* vol. 38, pp. 225-243, 1954.

[4] M. P. Rols and J. Teissié, "Electropermeabilization of mammalian cells. Quantitative analysis of the phenomenon. ," *Biophys. J. ,* vol. 58, pp. 1089-1098, 1990.

[5] H. J. Hinz, (Ed.), *Thermodynamic Data for Biochemistry and Biotechnology.* . Berlin and New-York: Springer-Verlag, 1986.

[6] E. B. Sozer, Z. A. Levine, and P. T. Vernier, "Quantitative Limits on Small Molecule Transport via the Electropermeome - Measuring and Modeling Single Nanosecond Perturbations," *Sci Rep,* vol. 7, p. 57, 2017.

[7] R. J. Ellis, "Macromolecular crowding: an important but neglected aspect of the intracellular environment," *Current Opinion in Structural Biology,* vol. 11, pp. 114-119, 2001.

[8] M. Kumar, M. S. Mommer, and V. Sourjik, "Mobility of cytoplasmic, membrane, and DNA-binding proteins in Escherichia coli," *Biophys J,* vol. 98, pp. 552-9, 2010.
